# Exploring tomato *Solanum pennellii* introgression lines for residual biomass and enzymatic digestibility traits

**DOI:** 10.1186/s12863-016-0362-9

**Published:** 2016-04-05

**Authors:** G. Caruso, L. D. Gomez, F. Ferriello, A. Andolfi, C. Borgonuovo, A. Evidente, R. Simister, S. J. McQueen-Mason, D. Carputo, L. Frusciante, M. R. Ercolano

**Affiliations:** Department of Agricultural Sciences, University of Naples ‘Federico II’, via Università 133, 80055 Portici, Italy; Department of Biology, Center for Novel Agricultural Products, University of York, Heslington, York YO10 5DD UK; Department of Chemical Sciences, University of Naples Federico II, via Cinthia 4, 80126 Naples, Italy

**Keywords:** *Solanum pennellii* population, Cell wall components, Saccharification, Biomass conversion, Extreme genotypes, Candidate genes

## Abstract

**Background:**

Residual biomass production for fuel conversion represents a unique opportunity to avoid concerns about compromising food supply by using dedicated feedstock crops. Developing tomato varieties suitable for both food consumption and fuel conversion requires the establishment of new selection methods.

**Results:**

A tomato *Solanum pennellii* introgression population was assessed for fruit yield, biomass phenotypic diversity, and for saccharification potential. Introgression lines 2–5, 2–6, 6–3, 7–2, 10–2 and 12–4 showed the best combination of fruit and residual biomass production. Lignin, cellulose, hemicellulose content and saccharification rate showed a wide variation in the tested lines. Within hemicellulose, xylose value was high in IL 6–3, IL 7–2 and IL 6–2, whereas arabinose showed a low content in IL 10–2, IL 6–3 and IL 2–6. The latter line showed also the highest ethanol potential production. Alkali pre-treatment resulted in the highest values of saccharification in most of lines tested, suggesting that chemical pretreatment is an important factor for improving biomass processability. Interestingly, extreme genotypes for more than one single trait were found, allowing the identification of better genotypes. Cell wall related genes mapping in genomic regions involved into tomato biomass production and digestibility variation highlighted potential candidate genes. Molecular expression profile of few of them provided useful information about challenged pathways.

**Conclusions:**

The screening of *S. pennellii* introgression population resulted very useful for delving into complex traits such as biomass production and digestibility. The extreme genotypes identified could be fruitfully employed for both genetic studies and breeding.

**Electronic supplementary material:**

The online version of this article (doi:10.1186/s12863-016-0362-9) contains supplementary material, which is available to authorized users.

## Background

Over the last decades, rising concerns upon depleting fossil fuels has resulted in an increased interest in fuels derived from bio-renewable sources including sugars, starch and lignocellulosic materials [1]. Lignocellulosic biomass materials constitute the most abundant renewable substrate for ethanol [2]. Currently, cellulosic feedstocks derived from dedicated biomass crops in the U.S., South America, Asia and Europe are from corn stover, sugarcane bagasse, or perennial low input-high yield crops such as miscanthus or switchgrass [3]. More interestingly, lignocellulosic biomass can be obtained in large-scale from agricultural residues, making their conversion into fuel more advantageous from the economic, environmental and strategic points of view [4]. In particular, the production of ethanol from biomass residuals represents a unique opportunity to avoid concerns about compromising food supply by using starch or sucrose based feedstocks [5–7]. So far, a few attempts have been conducted to investigate the potential of producing fuel from residual biomass of tomato (*Solanum lycopersicum* L.), a major vegetable crop worldwide.

Biomass production depends on several traits related to morphological and physiological processes controlling the plant vegetative growth. Developing new varieties for both food and fuel production, will require the establishment of new selection methods. Moreover, in order to elucidate genetic interactions between traits, it will be important to understand the correlations between traits and the extent to which they can be uncoupled, since positive and negative correlations can have profound effects on each other or comprising other aspects of crop production [8]. Genomic resources like wild introgression populations can facilitate the identification of tomato genotypes characterized by both high fruit and residual biomass production.

The tomato *Solanum pennellii* introgression population is a permanent mapping source for Quantitative Trait Loci (QTLs) analysis composed of a series of introgression lines, in which defined genomic segments of the *S. pennellii* genome replaced homologous regions in *S. lycopersicum* (cultivar M82) background. Such population can be very effective for identifying QTL, because any phenotypic difference between an introgression line and the recurrent parental line is attributed solely to donor parent genes within the introgressed chromosomal segment [9]. The assessment of *S. pennellii* introgression lines phenotypic and chemical traits can provide useful genetic information about tomato biomass production and potential fuel conversion.

Numerous structural and compositional features can have effects on lignocellulosic biomass processability. Cellulose is a polymer of 1–4 β linked glucose and forms crystalline fibrils within the cell walls. Hemicelluloses are complex-polymers of hexoses (mannose, glucose, galactose) and pentoses (xylose and arabinose), arranged in a non-crystalline manner, and interact with cellulose fibers [10]. The cellulose cristallinity and the heterogeneity of the hemicellulosic fraction confer recalcitrance to the biomass and represent a barrier for the utilization of the sugars locked in the polymers [11]. Pretreatments can be useful for modifying the architecture of the cell walls that compose the biomass, making it more accessible to hydrolytic enzymes. This involves the modification of lignin, removal of matrix polysaccharides, and reduction of cellulose crystallinity [12].

A way to improve feedstock amenability to fuel conversion is to delve into genetic variability within one species for breeding towards the best traits. In this work, we approach the study of a complex trait such biomass production and fuel conversion making use of *S. pennellii* introgression lines. Firstly, we assessed the tomato introgression lines biomass phenotypic diversity for identifying both traits contributing to high biomass yield and genotypes to employ in selection program for increasing biomass production. Then we characterised the residual biomass for lignin components, correlating them with the saccharification potential. In addition, we performed, on selected genotypes, a comparison among different cell wall polysaccharide components in order to identify chemical traits associated with tomato residual biomass digestibility. Finally, as proof of concept we assessed genes involved in cell wall biosynthesis in extreme genotypes by *in silico* analysis and by molecular assay, in order to investigate their role in phenotypic diversity.

## Results

### Characterization of morphological traits in S. pennellii introgression lines

**Fig. 1:**
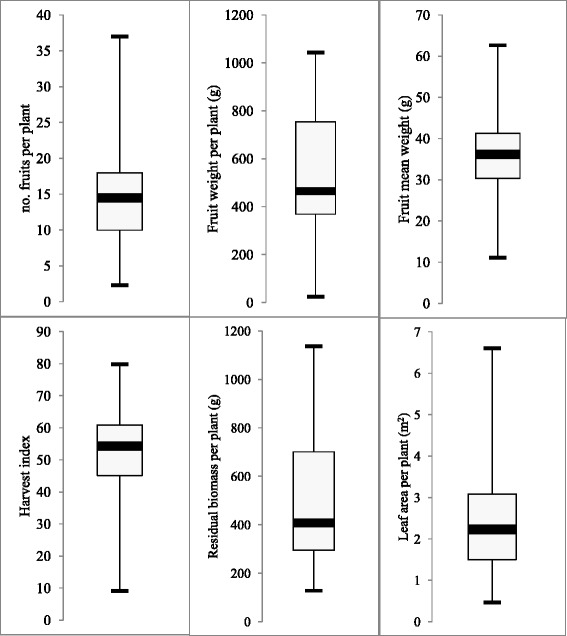
Frequency distribution of yield parameters, plant residual biomass and leaf area in 37 tomato introgression lines

Thirty-seven tomato introgression lines, able to cover the full *S. pennellii***genome**, have been assessed for fruit yield and biomass production. The frequency distribution of fruit yield and biomass parameters (Fig. 1) showed that the fruit yield of 50 % of the introgression lines felt in the range 369–754 g per plant, with a harvest index range of 45–61 and a residual biomass of 295–701 g. Overall, IL 12–4, 6–3 and 2–5 provided the highest fruit yields (1043, 978 and 958 g per plant respectively), whereas 4–4 and 6–2 gave the lowest (117 and 25 g per plant respectively), whilst the recurrent parent M82 showed a fruit yield of 665 g per plant and a harvest index of 61.5 (Additional file 1: Tables S1). Interestingly, IL 6-2 and IL 6–3 have both an indeterminate growth habit, but showed extremely different yield performance, in agreement with data reported by Eshed and Zamir [9].

For crop residual biomass and leaf expansion, IL 2–6 showed the highest values of 1136 g and 6.6 m^2^ per plant, respectively. It was followed by IL 4–4 (950 g) in the biomass ranking and by IL 12–4 (4.55 m^2^) with regard to leaf area. The lowest values of residual biomass and leaf area were showed by IL 3–2 and IL 1–1 (128 g and 0.47 m^2^ per plant respectively). M82 showed a biomass yield of 420 g per plant and a leaf area of 1.62 m^2^ per plant (Additional file 1: Tables S2 and Additional file 2: Table S3). Extreme genotypes were identified for each measured trait. The most skewed traits were fruit weight per plant, residual biomass per plant and leaf area per plant. The introgression lines 2–5, 2–6, 6–3, 7–2, 10–2 and 12–4 showed the highest combination of fruit and residual biomass production and the introgression lines 2–1, 3–2, 4–3, 5–1 and 7–1 the lowest.

### Lignin content and saccharification rate

**Fig. 2:**
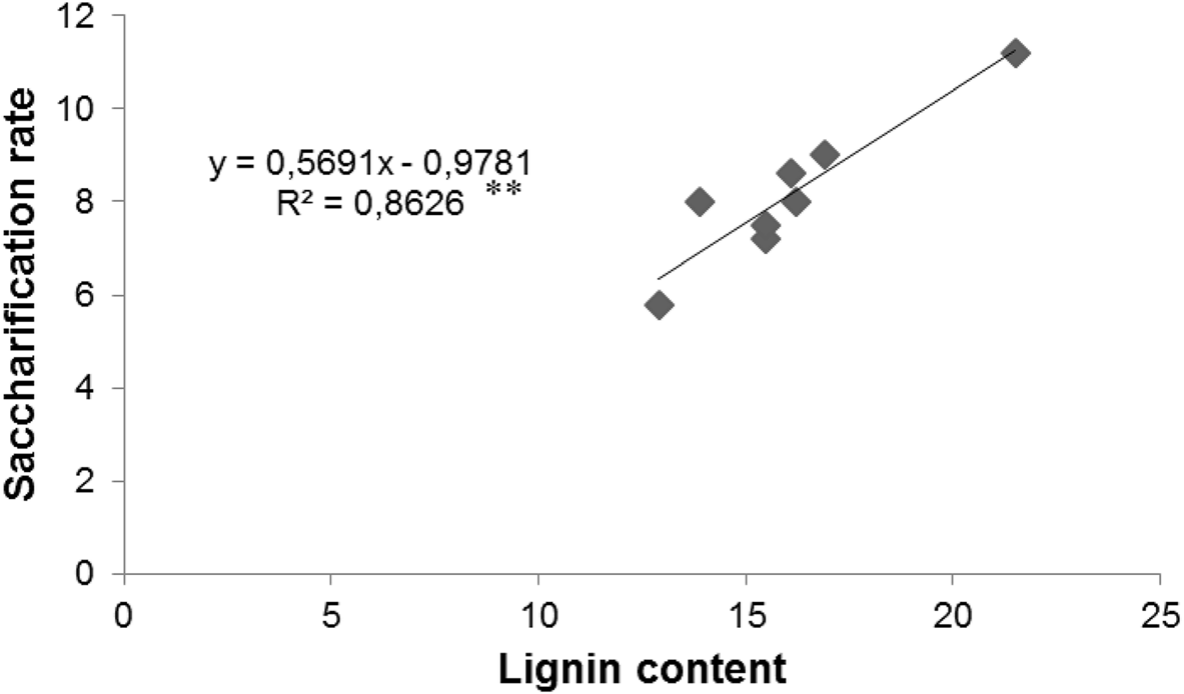
Linear regression between lignin content and saccharification rate in a subset of ILs with high dry biomass production

The highest lignin content was in IL 2-6 (21.5 g 100 g^−1^ of dry weight), the lowest in IL 1–1 and IL 8–3 (10.5 g 100 g^−1^), M82 display a lignin content of 17.6 g 100 g^−1^ of dry weight (Table 1; Additional file 2: Table S3). Saccharification rates higher than 10 mg glucose g^−1^ biomass h^−1^ (Table 1) were recorded in nine genotypes (IL 2–6, IL 3–3, IL 3–4, IL 4–3, IL 8–1, IL 8–2, IL 9–1, IL 10–2 IL 11-1 and M82), whereas a very low value was displayed by IL 8–3 (1.3 mg glucose g^−1^ biomass h^−1^). Fourty-seven per cent of genotypes evidenced a saccharification value ranging between 8 and 12 mg glucose g^−1^ biomass h^−1^. Only the 8 % of the introgression lines showed saccharification rates lower than 2 mg glucose g^−1^ biomass h^−1^ (IL 1–1, IL 3–2 and IL 8–3) and the 3 % higher than 13 mg glucose g^−1^ biomass h^−1^ (IL 3–4). Out of total lines analyzed, eight line, producing high amount of dry biomass (IL 1–3, IL 1–4, IL 2–2, IL 2–6, IL 6–1, IL 6–3, IL 7–2, IL 12–4), showed a correlation (R^2^ = 0.86) between saccharification rate and lignin content (Fig. 2).

**Table 1:** Lignin content and saccharification rate in 37 tomato introgression lines

Introgression lines	Lignin content g 100 g^−1^ dry weight		Saccharification rate mg glucose g^−1^ biomass h^−1^	
IL 1–1	10.5 ± 2.0	l	1.8 ± 0.4	il
IL 1–3	16.1 ± 1.3	ai	8.6 ± 0.2	ce
IL 1–4	15.5 ± 0.7	al	7.2 ± 0.7	dg
IL 2–1	13.4 ± 0.9	dl	5.9 ± 1.2	eg
IL 2–2	16.2 ± 0.7	ah	8.0 ± 1.0	cg
IL 2–5	12.0 ± 2.2	gl	2.7 ± 0.2	in
IL 2–6	21.5 ± 3.9	a	11.2 ± 2.6	ad
IL 3–1	15.1 ± 1.2	al	9.5 ± 0.8	kg
IL 3–2	10.6 ± 0.6	il	1.6 ± 0.3	l
IL 3–3	17.8 ± 0.5	ae	10.4 ± 1.4	ad
IL 3–4	16.4 ± 1.8	ai	13.4 ± 1.5	a
IL 3–5	13.1 ± 2.1	dl	5.6 ± 0.4	eh
IL 4–1	14.3 ± 3.7	cl	2.2 ± 0.6	in
IL 4–3	16.2 ± 1.6	ah	11.9 ± 2.3	ac
IL 4–4	14.6 ± 2.9	bl	5.9 ± 1.3	eg
IL 5–1	17.8 ± 1.4	ad	9.4 ± 1.6	bd
IL 5–2	12.3 ± 0.2	fl	2.5 ± 0.3	hl
IL 5–3	14.2 ± 3.3	cl	8.1 ± 0.6	cf
IL 6–1	12.9 ± 1.9	dl	5.8 ± 1.0	eg
IL 6–2	20.5 ± 2.1	a	7.7 ± 2.0	kg
IL 6–3	13.9 ± 0.4	cl	8.0 ± 2.3	ch
IL 7–1	14.9 ± 2.7	bl	5.2 ± 1.4	fh
IL 7–2	16.9 ± 4.3	ag	9.0 ± 1.0	ag
IL 7–4	16.1 ± 1.8	ai	4.7 ± 0.8	gi
IL 8–1	14.2 ± 1.8	cl	10.8 ± 0.4	ac
IL 8–2	18.4 ± 1.2	ad	12.0 ± 1.6	ab
IL 8–3	10.5 ± 1.4	l	1.3 ± 0.3	l
IL 9–1	13.2 ± 0.4	dl	10.2 ± 2.1	ad
IL 9–2	14.7 ± 2.7	bl	5.5 ± 2.0	eh
IL 9–3	10.8 ± 2.4	hl	8.2 ± 0.8	bh
IL 10–1	16.8 ± 1.4	ag	8.0 ± 1.6	cg
IL 10–2	14.5 ± 3.6	cl	10.3 ± 1.1	ae
IL 10–3	12.5 ± 1.7	el	2.0 ± 0.3	in
IL 11–1	16.8 ± 2.7	ag	10.6 ± 1.3	ac
IL 11–2	20.1 ± 3.6	ab	9.3 ± 0.3	bd
IL 11–3	20.0 ± 2.5	ab	8.5 ± 0.4	bh
IL 12–4	15.5 ± 4.3	al	7.5 ± 0.5	kg
M82 (control)	17.6 ± 2.7	af	12.0 ± 3.0	ab

Within each column, means followed by different letters are significantly

different according to the Duncan test at *p* ≤ 0.05

### Cell wall composition and theoretical ethanol yield

Table 2 shows the analysis of cell wall polysaccharide components performed on selected ILs. Total cellulose ranged from 40.7 g 100 g^−1^ of dry weight (IL 4–1) to 55.9 g 100 g^−1^ of dry weight (IL 4–3). Within the total cellulose, the crystalline fraction accounted for 3.5 (IL 3–1) to 17.6 (IL 4–3) g 100 g^−1^ of dry weight. The highest hemicellulose content recorded in IL 4–3 (6.0 g 100 g^−1^ d.w.) was about three times greater than that showed by IL 2–5. Finally, the pectin content displayed a wide range comprised between 2.0 and 10.1 g 100 g^−1^ d.w. (in IL 9–3 and IL 10–3 respectively). M82 showed a low level of total cellulose (38.7 g 100 g^−1^ of dry weight) but the crystalline fraction and hemicellulose accounted for 15.2 and 5.2 g 100 g^−1^ of dry weight respectively. Among the introgression lines tested, IL 2–6 and IL 6–3 showed high content of cellulose (51.7 and 55.8 g 100 g^−1 ^d.w. respectively) and hemicellulose (5.6 and 4.4 g respectively) but moderate crystalline cellulose (11.5 and 12.5 g). Conversely, IL 3–1 and IL 4–1 showed low content of cellulose (41.0 and 40.7 g 100 g^−1 ^d.w. respectively), crystalline cellulose (3.5 and 5.4 g respectively) and hemicellulose (2.9 and 2.5 g respectively). Interestingly, lignin was positively correlated with crystalline cellulose (*r* = 0.52 at *p* < 0.05) and with hemicellulose (*r* = 0.51 at *p* < 0.05). Crystalline cellulose, in turn, was also correlated with hemicellulose (*r* = 0.87 at *p* < 0.01).

**Table 2:** Chemical composition of 13 tomato introgression lines residual biomass

Introgression lines	Total cellulose g 100 g^−1^ of dry weight		Crystalline cellulose		Hemicellulose		Pectin	
IL 2–5	51.5 ± 2.3	c	4.6 ± 0.8	de	1.9 ± 0.3	g	7.3 ± 0.3	c
IL 2–6	51.7 ± 3.1	c	11.5 ± 2.4	ad	5.6 ± 1.5	ab	5.8 ± 0.5	d
IL 3–1	41.0 ± 1.9	fg	3.5 ± 1.0	e	2.9 ± 1.2	dg	2.6 ± 0.7	i
IL 4–1	40.7 ± 2.1	fg	5.4 ± 1.2	ce	2.5 ± 0.4	fg	5.2 ± 0.3	ef
IL 4–3	55.9 ± 2.2	a	17.6 ± 2.6	a	6.0 ± 0.9	a	3.4 ± 0.6	h
IL 6–2	44.6 ± 2.3	e	13.2 ± 2.6	ab	4.0 ± 0.9	cf	5.1 ± 0.4	ef
IL 6–3	55.8 ± 1.9	a	12.5 ± 2.4	ac	4.4 ± 1.5	bd	2.1 ± 0.7	i
IL 7–2	41.6 ± 1.7	fg	14.2 ± 2.5	ab	4.2 ± 1.4	be	3.2 ± 0.6	h
IL 9–3	48.2 ± 3.3	d	9.4 ± 1.3	be	4.1 ± 0.4	bf	2.0 ± 0.3	i
IL 10–2	46.1 ± 2.2	e	11.0 ± 2.2	ae	4.8 ± 0.4	ac	5.6 ± 0.5	de
IL 10–3	42.4 ± 2.4	fg	4.8 ± 1.1	de	2.8 ± 0.4	dg	10.1 ± 0.5	a
IL 11–3	50.1 ± 2.5	c	13.9 ± 2.9	ab	4.1 ± 0.3	be	4.6 ± 0.1	fg
IL 12–4	53.6 ± 2.9	b	3.6 ± 0.8	e	2.7 ± 0.4	eg	9.3 ± 0.3	b
M82 (control)	38.7 ± 3.0	g	15.2 ± 3.4	ab	5.2 ± 1.0	ac	4.0 ± 0.2	g

Within each column, means followed by different letters are significantly different according to the Duncan test at *p* ≤ 0.05

**Fig. 3:**
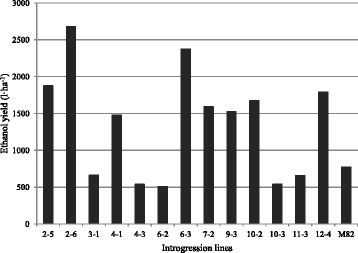
Theoretical ethanol yield in 13 tomato introgression lines plus M82

The monosaccharide composition in the hemicellulosic fraction showed a large degree of variability between genotypes tested (Table 3). Glucose was the highest represented sugar, ranging from 26.9 g 100 g^−1^ d.w (IL 4–1) to 18.4 g 100 g^−1^ d.w (IL 4–3). Xylose followed glucose in the rank, showing a variation comprised between 13.4 g 100 g^−1^ d.w. in IL 3–1 and 26.5 g 100 g^−1^ d.w in IL 6–3. Fucose was the least represented monosaccharide, with values ranging from 0.27 (IL 2–6) and 0.92 g 100 g^−1^ d.w. (IL 10–3). Based on cell wall content of cellulose and hemicellulose monosaccharides (on dry weight) a theoretical ethanol yield was calculated (Fig. 3). IL 2–6 showed the highest ethanol potential production (2681 L ha^−1^) as it provided with the highest biomass yield (6 t ha^−1^) and a good conversion of biomass into ethanol (451 L t^−1^ d.w.). IL 4–3 resulted in a very low theoretical ethanol yield (539 L ha^−1^), in spite of the best biomass quality for ethanol production (486 L t^−1 ^d.w.). Finally, IL 4–1 showed the lowest potential conversion of biomass into ethanol (340 L t^−1^ d.w).

**Table 3:** Monosaccharide composition of hemicellulose in 13 tomato introgression lines

IL	Ara		Fuc		Gal		Gal A		Glc A		Glc		Man		Rha		Xyl	
	g 100 g^−1^ hemicellulose																	
IL 2–5	11.6	ad	0.41	b	20.1	ce	3.3	ef	0.85	c	25.6	a	12.6	ab	6.0	bf	19.5	be
IL 2–6	10.0	e	0.27	c	16.9	g	10.0	ab	1.98	ab	20.5	bd	12.1	ad	5.6	cf	22.6	ad
IL 3–1	13.6	a	0.37	bc	23.3	a	5.5	ce	1.64	ac	23.6	ab	11.2	be	7.4	a	13.4	f
IL 4–1	13.0	ab	0.90	a	20.4	bd	2.4	f	1.41	ac	26.9	a	10.5	e	6.7	ac	17.9	df
IL 4–3	12.0	ad	0.28	c	18.0	eg	8.3	bc	1.66	ac	18.4	d	12.4	ac	5.7	bf	23.1	ad
IL 6–2	12.4	de	0.34	bc	18.9	g	6.4	cd	1.12	bc	19.1	d	12.1	ad	5.2	ef	24.5	ac
IL 6–3	10.5	ad	0.36	bc	16.8	cg	7.2	bc	1.86	ab	18.5	cd	13.2	a	5.1	df	26.5	a
IL 7–2	12.0	ad	0.36	bc	18.1	eg	7.0	bc	1.47	ac	19.7	bd	11.6	be	5.1	ef	24.7	ab
IL 9–3	11.4	be	0.33	bc	20.7	bc	9.6	ab	1.76	ac	19.9	bd	11.3	be	5.7	bf	19.5	be
IL 10–2	10.6	de	0.33	bc	18.1	eg	11.7	a	2.35	a	21.1	bd	10.5	de	6.2	be	19.1	ce
IL 10–3	12.9	ac	0.92	a	19.9	cf	3.7	df	1.25	bc	23.0	ac	8.9	f	6.8	ab	22.7	ad
IL 11–3	10.9	ce	0.42	b	17.8	fg	7.2	bc	1.77	ac	22.6	ad	12.8	ab	4.9	f	21.6	ad
IL 12–4	12.7	ac	0.32	bc	22.5	ab	5.4	ce	1.16	bc	26.1	a	11.3	be	6.3	bd	14.3	ef
M82	11.2	be	0.28	c	18.4	dg	9.7	ab	1.63	ac	21.3	bd	10.8	ce	5.9	bf	20.7	bd

Within each column, means followed by different letters are significantly different according to the Duncan test at *p* ≤ 0.05

*IL* introgression lines, *Ara* arabinose, *Fuc* fucose, *Gal* galactose, *Gal A* galacturonic acid, *Glc A* glucuronic acid, *Glc* glucose, *Man* mannose, *Rha* rhamnose, *Xyl* xylose

### Comparison among different saccharification pretreatments

**Fig. 4:**
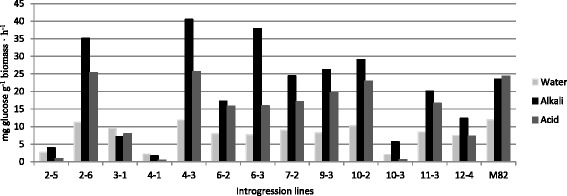
Saccharification rate in 13 tomato introgression lines plus M82, as a function of the pretreatment type

Three biomass pretreatments were assessed in order to understand if saccharification rate is affected by treatment in genotype specific manner. Alkali pre-treatment caused the highest values of saccharification in most lines, whereas water pre-treatment was generally the least effective (Fig. 4). IL 4–3, IL 6–3, and IL 2–6 resulted in the highest values of saccharification rate (41, 38 and 35 mg glucose g^−1^ biomass h^−1^ respectively) using the alkali pretreatment. Interestingly, the saccharification rate was positively correlated with cellulose content, upon alkali pre-treatment, and with the crystalline cellulose content and hemicellulose content upon all the three pretreatments tested (Additional file 3). Finally, IL biomass saccharification rate was not significantly correlated with the hemicellulose arabinose/xylose ratio, regardless of the pre-treatment type. Our results display that biomass enzymatic digestibility is affected both by genotype cell wall structures and pretreatment type employed.

### In silico identification of candidate QTL involved in biomass production

Based on information deposited in cell-wall literature studies and in database resources [13], we selected 23 protein families implicated in the construction and modification processes of plant cell walls. Using this dataset, the tomato genome was explored to identify the enzymes involved in the biosynthesis of key polysaccharides such as pectin, lignin, cellulose, hemicellulose and starch. In order to identify candidate QTL for biomass production by *in silico* analysis, we looked at selected ILs for cell wall related genes (see Additional file 2). Several genes located in the introgression regions of contrasting genotypes 2–5, 2–6, 3–1, 4–1 4–3, 6–2, 6–3, 7–2, 9–3, 10–2, 10–3, 11–3, and 12–4 were identified. Interestingly, introgression region 2–6 included a high number of Peroxidases, Laccases, Glycosyltransferases and MYB transcription factors in a region of 3.7 Mb. Moreover, two Cellulose synthase, two UDP-D-glucose dehydrogenase, a specific GDP-mannose dehydratase, a Sucrose synthase, a Galactosyltransferase, a GDP-mannose dehydratase and a GHMP kinase have been identified in this chromosome area (Table 4). Conversely, IL 4–3 in a much larger region includes only 30 genes coding for enzymes involved in cell wall biosynthesis.

**Table 4:** Proteins involved in the construction and modification processes of cell wall polysaccharides identified in tomato 13 introgression line chromosome regions

Line	Region size MB	CS n. ^a^	Glt n.	Per n.	Lac n.	Ald n.	Ino n.	MY n.	Akr n.	Ugd n.	Dat n.	Gat n.	Gph n.	Gdd n.	Ss n.	GHk n.
IL 2–5	4.3	1	3	3	1			5								
IL 2–6	3.7	2	6	20	7	–	1	19		2	–	1		1	1	1
IL 3–1	3.7	1	3	3				1								
IL 4–1	3.1	1	1					1								
IL 4–3	58.9	2	2	5	5	3		12					1			
IL 6–2	8.2					2	1	8		1						
IL 6–3	2.8		3	2	5			6								
IL 7–2	7.7		4	5	2			3								
IL 9–3	4.6	1	1					2	2					1		
IL 10–2	5.8	1	1	8	2	1		9			1					
IL 10–3	1.6	1		2	1	1		4			1					
IL 11–3	21.2	2	7	1	2			3			1					
IL 12–4	3.7	1	1	2	2	1	1	8								

^a^ number of genes included in the chromosome region indicated

*CS* Cellulose synthase, *Glt* Glycosyltransferase, *Per* Peroxidase, *Lac* Laccase, *Ald* Alcohol dehyd, *Ino* Inositol Oxygenase, *My* MYB Trascription Factor, *Akr* Aldo/ketoreductase, *Ugd* UDP-D-glucose dehydrogenase, *Dat* 3-deoxy-D manno-octulosonic acid transferase, *Gat* Galactosyltransferase, *Gph* Galactose phosphatase, *Gd* GDP-mannose dehydratase, *Gut* Glucosyltransferase, *Ss* Sucrose synthase, *GHk* GHMP kinase

### Molecular expression profile

**Fig. 5:**
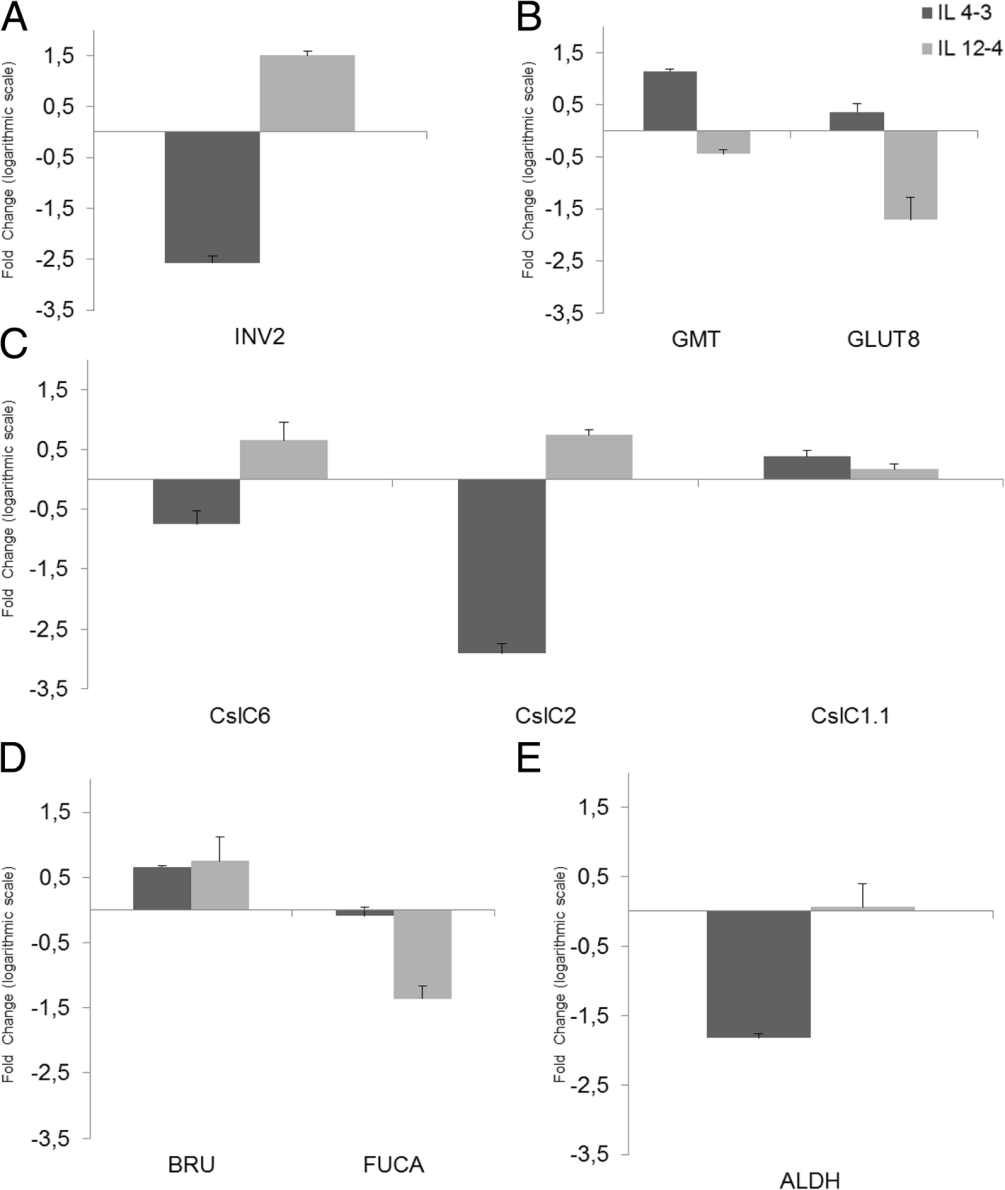
Expression fold change of different genes among IL 12-4 (*dark gray*) and IL 4-3 (*light gray*) respect to M82. Panel (**a**) shows the expression of cell wall invertase (INV2); (**b**), GDP-mannose transporter (GMT) and Glucose transporter 8 (GLUT8); (**c**), cellulose synthase-like glycosyltransferase (CslC1-1,CslC2, CslC6); (**d**),  xyloglucan endotransglucosylase (BRU1) and α-L-fucosidase1 (FUCA1); and (**e**), Aldehyde dehydrogenase (ALDH). Error bars relative to RT–qPCR experiments represent the estimate of standard error of the mean (SEM) for the replicates

RT- qPCR analysis was used to analyze the expression pattern of key genes in two contrasting ILs (12–4 and 4–3). Aldehyde dehydrogenase (ALDH), Xyloglucan endotransglucosylase (BRU1), Cellulose synthase-like glycosyltransferase (CslC1-1,CslC2, CslC6), Alpha-L-fucosidase1 (FUCA1) Glucose transporter 8 (GLUT8), GDP-mannose transporter (GMT) and Cell-wall invertase (INV2) gene expression were assessed in the two selected lines and in the M82 genotype used as control. The invertase INV2 gene showed an opposite expression pattern in the two ILs tested, in fact it was up-regulated in IL 12-4 and down-regulated in IL 4-2 (Fig. 5a). GMT and GLUT8, respectively mannose and glucose transporters showed an increased transcription in IL 4–3 compared to M82 control, while were both down regulated in IL 12–4 (Fig. 5b). The expression of the cellulose synthesis like genes was up-regulated in IL 12–4 and down-regulated in IL 4–3, except for the Cslc1.1 gene (Fig. 5c). Transcription levels of the BRU gene, involved in xyloglucan synthesis, was high in both IL, while transcription of FUCA, involved in hydrolysis L-fucose, was lower than in M82 in both ILs (Fig. 5d). Finally, the expression of ALDH was slightly up-regulated in IL 12–4 and down-regulated in IL 4-3 (Fig. 5e).

## Discussion

Large variations in fruit yield and biomass production among lines was evidenced in a two years field experiment conducted with *S. pennellii* IL population. Our results support previous findings [14] reporting that most of tomato introgression lines showed lower yield and fruit mean weight but higher vegetative biomass than the parent M82. Interestingly, in our study a number of genotypes with extreme phenotypes were identified. Analysis of extreme genotypes that exceed +/− 2.5 mean value of quantitative trait loci (QTL), provide nearly equivalent power to complete genotyping at a reduced cost [15, 16].

The introgression lines tested exhibited dramatic variations also in cell wall lignin content, with the highest value being more than two times greater than the lowest. A similar survey on a set of *Arabidopsis thaliana* mutants for lignin biosynthetic genes revealed that lignin content ranged from 6.0 g 100 g^−1^ dry weight to 15.9 [17]. Lignin composition could affect the saccharification efficiency and usually plants with increased biomass and reduced lignin have an improved fuel production [17]. However in our work a subset of ILs with high dry biomass and lignin content, also showing a high saccharification rate was identified. Different Mischanthus genotypes also display different patterns of correlation between lignin content and saccharification efficiency [18]. Transgenic plants with modified lignin composition enhanced biomass saccharification [19]. In tobacco, up-regulation of sucrose metabolism genes appears to directly impact primary growth and therefore biomass production, also with slight decrease of lignin content [20]. Determining sample composition, especially structural carbohydrate, and using this information to predict relative performance for fuel conversion could be very useful [21]. In our study a number of lines with high potential ethanol production were identified, and as outlined, ethanol production can depend on different cell wall structure variations. Indeed, the cell wall architecture can be assembled from many different types of polysaccharides, phenylpropanoids and structural proteins. In our research, crystalline cellulose was highly correlated with hemicelluloses; with this respect, the amount and composition of branches attached to the hemicellulose backbone can affect the cell wall plasticity and crystal structure [22]. Within the hemicelluloses, the amount of xylan was high in IL 2–6, IL 4–3, IL 6–2, IL 6–3, IL 7–2, IL 10–3 and IL 11–3 and low in IL 3-1 and IL 12–4. Cell wall recalcitrance varies among plant species and even within different genotypes of the same species [23]. The close relationship between recalcitrance and the chemical composition of the non cellulosic matrix suggests that cell wall strength could be tuned by carefully controlling the matrix composition [17, 24].

Knowledge on cell wall composition can be useful for better direct genetic approaches and pretreatment design to render biomass more amenable to processing. In this respect, the effect of lignin as well as of cellulose on biomass digestibility has been described in previous research [25]. As for the effect of pretreatment on biomass saccharification rate, in our research alkali pretreatment showed the best performances. Consistently with our results, Lima et al. [26] reported that one-step alkali pretreatment improves the enzymatic digestibility of Eucalyptus bark compared to two-step pretreatment with HCl 1 % followed by 4 % NaOH. Acid and alkali pretreatments have distinct mechanisms for biomass modification [27]. Acid pretreatment involves the hydrolysis of hemicelluloses by breaking the glycosidic linkages of polysaccharides [28]. Alkali pretreatment, in turn, breaks down the intermolecular ester bonds that cross-link lignin with hemicelluloses, thereby solubilizing lignin and hemicelluloses [29]. The removal of hemicelluloses increases the mean pore size of the substrate, which facilitates the hydrolysis of cellulose [30]. Correlation among parameters can be affected by composition. Significant positive correlation between hexose sugar production and hemicellulose arabinose content or arabinose/xylose ratio resulted in enhanced biomass digestibility both with acid and alkaline pre-treatments [31]. The composition study conducted here allowed us to detect genotypes with wide differences in biomass production, as well as in cell wall composition. Such large differences in the composition of biomass have been previously observed by van Acker et al. [17] in both, cellulose and hemicellulose glucose content among Arabidopsis thaliana mutants. A similarly large variation was also observed by Marriott et al. (2014) in Brachypodium mutants. The variation in biomass composition between lines observed in the present work, although surprisingly wide, can be explained by the fact that these ILs originated from a genetically divergent biparental population. The identification of genes that affect such traits is still challenging. To this purpose a core panel of genes involved in metabolic pathways of cell wall biosynthesis and degradation was mapped in selected ILs with extreme phenotypes. These candidate genes encode proteins predicted to play a role in the synthesis, modification, assembly and disassembly of lignin, hemicellulose, cellulose and pectin [32]. A previous comparative approach showed that the differences in wall architecture between Arabidopsis and rice actually mirror the diversity of the individual gene families involved in the cell wall dynamics of the respective plant species [33]. Changes in wall composition or architecture could be due to mutations either in genes directly related to cell wall metabolism or in genes involved in regulation [34]. Morgan et al. [35] performed a biochemical analysis of a tomato introgression line with increased levels of fruit citrate identifying a target gene that was successfully tested in transgenic plants. Mutations with major effect more frequently occur in domesticated or artificially disturbed populations [36]. This supports the use of inbreds obtained between wild and domesticated species for identifying traits with strong and repeatable phenotypes [37]. In our work several specific candidate genes co-localizations could be underlined. For example, the genomic region delimitated by IL 2–6 contains 20 Peroxidase, 19 MYB factors, 6 Glycosyltransferase, 7 Laccase, 2 UDP-D-glucose dehydrogenase and 2 Cellulose synthase as well as other important polysaccharides biosynthesis enzymes. Different isoforms of UDP-glucose-consuming pathway have a regulatory role in carbon partitioning between cell wall formation and sucrose synthesis [38]. A Glycosyltransferase mutant produced plants deficient in ferulic and coumaric acid, aromatic compounds known to be attached to arabinosyl residues in xylan substituted with xylosyl residues. The mutant plants exhibit an increased extractability of xylan and increased saccharification, probably reflecting a lower degree of diferulic cross-links [39]. Genes and regulatory elements present in *S. pennelli* and *S. lycopersicum* could be involved in alterations of cell wall composition and biomass production.

The molecular expression profile suggests that the partioning between source and sink organ could be challenged in different lines. Indeed, IL 12–4 (a high biomass producer line) showed a major cleavage of sucrose compared to IL 4-3, due to the up-regulation of a cell wall invertase (INV2) and a lower activity of a GDP-mannose transporter and a of glucose transporter 8. Cleavage of sucrose by invertase is generally correlated with growth and cell expansion, associated with sucrose partioning [40]. Excess of sucrose is broken down into fructose and UDP-glucose, which is employed in the synthesis of cell wall polymers [20]. Cellulose synthase-like (CslC2 and CslC6) proteins, involved in the synthesis of various β-glycan polymers [41] by using GDP-mannose as substrate, result both up-regulated in IL 12-4 and down-regulated in IL 4-3 (low biomass producer line) whilst CslC1 is up-regulated in both. A decrease of mannose substrate to synthesize 1,4-β-glucan backbone of mannose could improve IL12-4 digestibility. The expression of ALDH, an enzyme involved in the synthesis of lignin components such us ferulate and sinapate [42] is also up-regulated in IL 12-4. Both ILs have a similar amount of lignin but different saccharification rate; this difference may be related either to their lignin composition or to the fact that saccharification is a multigenic trait which is affected by a large number of factors in the cell wall [43]. Genes involved in carbon metabolism mapped in IL extreme genotypes are part of a “network QTL”, where several elements of a metabolic network are affected by expression QTLs, enzyme activity QTLs, or metabolite QTLs [44, 45] fine tuned in any genotype.

## Conclusions

The present work shows the potential for exploitation of tomato ILs residual biomass for fuel conversion. The tomato introgression lines showed high variability in biomass production, cell wall composition and saccharification rate and, consequently, potential ethanol yield also resulted in a wide range of values among the genotypes. The trait enhancement found in extreme genotypes could be compared with the behavior of recurrent parent through a combined genomic and chemical profiling. It is evident, even from literature, that an intricate network of relations among components govern biomass production and digestibility. For this reason it is difficult to identify genotypes that combine both characteristics, on the other hand expression pattern of genes related to biomass production provided interesting clues which should be further investigated. Precise gene mapping is needed in order to predict biomass quality based on genomic information, while the interrogation of contrasting lines could permit the identification of the most eligible alleles linked to saccharification efficiency. Indeed, selected lines will be further explored to identify the candidate genes involved.

## Methods

### Phenotypic analysis

Research was carried out for two years in Naples, southern Italy (40°50' N, 14°15' E, 17 m a.s.l.), on the commercial tomato variety M82 (*Solanum lycopersicum* L.), *Solanum pennellii* LA716 and on 37 *Solanum pennellii* tomato introgression lines, kindly provided by Dr Dani Zamir (Hebrew University of Jerusalem) and reported in Table 1. Information on such lines can be found at website http://zamir.sgn.cornell.edu. A randomized complete block design with three replicates was arranged and each plot had a 4.73 m^2^ (1.75 x 2.70 m) surface area. Transplanted plants were arranged in single rows spaced by 0.90 m from each other and the spacing was of 30 cm along the rows (3.2 pt · m^−2^). Experimental research on plants were conducted in accordance with local legislation. Plants were grown under standard tomato field procedures used for the area and fruits were harvested at full ripening. *S. pennellii* plants failed to grow properly in our climatic conditions and the few samples obtained were not included in the following analysis.

### General analytical methods

Plant samples were randomly selected to assess the maximum leaf surface extension using a bench top LI-COR leaf area meter. At harvest, the following determinations were made: a) weight and number of ripe undamaged fruits, classified as marketable; fruit mean weight on 50 unit samples; b) residual biomass, including leaves, shoots, stems; and c) immature or damaged fruits. Harvest index was calculated as a ratio between marketable fruits and total plant weight. After harvest, residual biomass showed no fungal symptoms; therefore samples were randomly collected in each plot and immediately transferred to the laboratory, where they were dried in an oven at 70 °C under vacuum until they reached constant weight. After assessing the dry residue, samples were carefully milled, avoiding mixing of materials belonging to different plant organs. The final material, composed of particles ≤ 1 mm diameter, was stored in air-tight bags at −20 °C and further dried just before being processed.

### Chemical analyses

After harvesting and weighing out, the residual plant biomass collected was oven-dried at 60 °C. Lignin determination and saccharification assay using water pre-treatment were performed in all the 38 genotypes tested for yield and biomass production. Other analyses (lignin, cellulose, hemicellulose, pectin, hemicellulose monosaccharides, crystalline cellulose and saccharification assay with acid or alkaline pretreatment) were performed on 13 selected genotypes (5 genotypes with biomass production < 300 g pt^−1^ of fresh weight; 3 genotypes with biomass production from 301 to 800 g pt^−1^ of fresh weight; 5 genotypes with biomass production > 800 g pt^−1^ of fresh weight).

### Lignin determination: acetyl bromide method

Biomass powder was weighed out (4 mg) into 2 mL tubes. The biomass was heated at 50 °C for 3 h after adding 250 μL of acetyl bromide solution (250 μL of acetyl bromide and 750 μL of glacial acetic acid in volume) and vortexing every 15 min. After the samples were cooled to room temperature, the content was transferred into 5 mL volumetric flasks. A further 1 mL of NaOH (2 mol L^−1^) was used to rinse the tubes pouring the NaOH into the 5 mL flasks. 175 μL of hydroxylamine HCl (0.5 mol L^−1^) was added to the volumetric flasks and, after vortexing, the latter were filled up to 5 mL with glacial acetic acid and mixed several times. Finally, in order to measure the 280 nm UV adsorption by spectrophotometer, 100 μL of each sample was diluted in 900 μL of glacial acetic acid. The amount of lignin was calculated using the following formula: $$ \begin{array}{l}\left[\mathrm{absorbance}/\left(\mathrm{coefficient}\ \mathrm{pathlength}\right)\right]\ \left[\left(\mathrm{total}\ \mathrm{volume}\ 100\%\right)/\mathrm{biomass}\ \mathrm{weight}\right],\ \mathrm{where}\ \mathrm{coefficient} = \\ {}15.69,\ \mathrm{pathlength} = 1,\kern0.2em \mathrm{total}\ \mathrm{volume} = 5,\ \mathrm{biomass}\ \mathrm{weight} = 4.\end{array} $$

### Cellulose, hemicellulose and pectin determination

#### Holocellulose

A mixture of 240 mL of water, 0.75 mL of glacial acetic acid and 2.25 g of sodium chlorite were added to 7.5 g of extracted and dried sample and kept at 75 °C for 3 h.

At hourly intervals, a volume equivalent to the initial amounts of glacial acetic acid and sodium chlorite was added to the biomass. The sample obtained was filtered and washed up first with cold water, then with warm water and finally with acetone. The residue was oven-dried at 105 °C for 24 h and then weighed to calculate the content of holocellulose.

#### Pectin

1.3 g amount of the resulting holocellulose was treated with 26 mL of potassium acetate (0.6 mol L^−1^) and incubated at 75 °C for 3 h before adding 26 mL of ammonium oxalate (0.04 mol L^−1^). The suspension was kept at 75 °C for 3 h. Then, the samples were filtered and washed up with excess of water before the residue was oven-dried at 105 °C for 24 h. The pectin content was calculated as the difference between the holocellulose fraction and the above residue.

#### Cellulose and hemicellulose

A sample of holocellulose (3.8 g) was treated with 100 mL of sodium hydroxide (4.4 mol L^−1^) at room temperature for 30 min and filtered. Then, it was washed up sequentially with warm water (200 mL), 5 mL of acetic acid (2 mol L^−1^) and 500 mL of water. Next, the residue was oven-dried at 105 °C for 24 h and weighed, providing the cellulose fraction. The hemicellulose content was calculated by subtracting the cellulose and pectin amount from that of holocellulose. The filtration process as well as the subsequent residue drying reported for pectin, cellulose and hemicellulose were accurately performed and, in fact, neither material loss nor different water content in the dried cell walls compared to the initial samples were assessed.

### Non cellulosic monosaccharide determination

Biomass dry powder (4 mg) was partially hydrolyzed by adding 0.5 mL of trifluoroacetic acid (2 mol L^−1^). Then, the vials were flushed with dry argon, mixed and heated at 100 °C for 4 h, mixing periodically. The vials were then cooled to room temperature and dried in centrifugal evaporator with fume extraction overnight. The pellets were washed twice with 500 μL of 2-propanol and vacuum dried. Finally, the samples were resuspended in 200 μL of deionised water, filtered with 0.45 μm PTFE filters, and analyzed by HPAEC. Monosaccharides were separated and quantified by HPAEC using a Dionex ICS-3000 with integrated amperometry detection. Chromatographic separation was performed on a CarboPac PA20 (3 x 150 mm) column (Thermo) using a gradient elution. The mobile phase consisted of solution A: 100 % water, solution B: 200 mM NaOH, and solution C: 0.1 M Sodium Hydroxide, 0.5 M Sodium Acetate. A flow rate of 0.5 ml min-1 was used and the gradient was as follows: 0 min: 100 % A; 5 min: 99 % A, 1 % B; 15 min: 99 % A, 1 % B; 22 min: 47.5 % A, 22.5 % B, 30 % C; 30 min: 47.5 % A, 22.5 % B, 30 % C. The column was then washed as follows: 30.1 min: 100 % B; 37 min: 100 % B; 37.1 min: 99 % A, 1 % B; 50 min: 100 % A; 55 min: 100 % A. The separated monosaccharides were quantified by using external calibration with a mixture of nine monosaccharide standards at 100 μM (arabinose, fucose, galactose, galacturonic acid, glucose, glucuronic acid, mannose, rhamnose, and xylose) that were subjected to acid hydrolysis in parallel with the samples.

### Crystalline cellulose

Biomass dry pellets after TFA hydrolysis were washed once with 1.5 ml of water, and twice using 1.5 ml of acetone. The dried pellets were left to air dry overnight before complete hydrolysis by adding 90 μl of 72 % (p/v) sulfuric acid, incubating at room temperature for 4 h. 1.89 ml of water was subsequently added and the sample was heated for 4 h at 120 °C. The glucose content of the supernatant was assessed using the colorimetric Anthrone assay, using a glucose standard curve.

### Theoretical ethanol yield calculation

The theoretical ethanol yield was calculated considering the total cellulose conversion in the sample, according to the National Renewable Energy Laboratory standards [46, 47]. The theoretical ethanol yield was expressed also taking into account agronomical traits such as the biomass yield per surface area unit.

### Saccharification assay

#### Formatting of plant materials

Loading of plant powder into 96-well plates, using a custom-made robotic platform (Labman Automation, Stokesley, North Yorkshire, UK), and saccharification assays were performed according to Gomez et al. (2010) [48] after water, acid or alkali pretreatment. Enzymatic hydrolysis was carried out using an enzyme cocktail with a 4:1 ratio of Celluclast and Novozyme 188.

### In silico search for cell wall related genes

Marker sequence coordinates that delimitated introgression lines (2–5, 2–6, 3–1, 4–1, 4–3, 6–2, 6–3, 7–2, 9–3, 10–2, 10–3, 11–3, 12–4) were downloaded by SGN website [13]. ITAG annotated proteins retrieved in the genomic areas bounded by the markers chosen were recorded in an excel file. Proteins involved in the construction and modification processes of cell wall polysaccharides (Additional file 2) have been searched for each IL line genomic area and stored in a separated file.

### Gene expression analysis

Total RNA of IL 12–4, IL 4–3 and M82 genotypes was extracted from leaf tissues, using a Kit Spectrum plant total RNA (Sigma), and treated with DNase I Digestion (Sigma). Total RNA was quality checked and cDNA synthesis was performed with oligo (dT) and SuperScript III Reverse Transcriptase (Invitrogen). Specific primers for candidate genes (Additional file 2: Table S5) were designed using Primer3 software. RT–qPCR was performed in a 12.5 μL reaction volume using the SensiFAST SYBR Hi-ROX Kit (Bioline) with 4.5 μL cDNA as a template. Each reaction was carried out in triplicate and run on the 7900HT Fast Real-Time PCR System (Applied Biosystems). Fold change of each transcript, normalized to EF (elongation factor), was calculated relative to expression in the M82 sample, using the 2^–ΔΔCt^ method.

### Statistical analysis

Data were processed by analysis of variance and mean separations were performed through the Duncan multiple range test, with reference to 0.05 and 0.01 probability levels, using SPSS software version 17. Data expressed as percentage were subjected to angular transformation before processing. Correlations were performed with all pairs of chemical parameters using the software mentioned above.

## Availability of data and materials

Supporting data are included as additional files.

## Additional files

Additional file 1: Table S1. Fruit yield and harvest index of 37 tomato introgression lines. **Table S2.** Residual biomass and leaf area of tomato introgression lines. (DOCX 113 kb) Additional file 2: Table S3. ANOVA analysis on phenotypic and chemical traits. **Table S4.** Protein families involved in the construction and modification processes of cell wall polysaccharides. **Table S5.** Tomato cell wall related proteins retrieved in *S. pennelli* genome region of selected introgression lines. **Table S6.** List of primers used in qPCR assay. (XLSX 55 kb) Additional file 3: Figure S1. Correlation between saccharification rate and crystalline cellulose and between saccharification rate and hemicellulose in 13 tomato introgression lines plus M82. (PDF 13 kb)
