# Supplementary material for: Exploring tomato Solanum pennellii introgression lines for residual biomass and enzymatic digestibility traits
Source: BMC Genet. 2016 Apr 5;17:56. doi: 10.1186/s12863-016-0362-9 (PMC4820949; doi:10.1186/s12863-016-0362-9)
Supplement: Additional file 1: Table S1. — Fruit yield and harvest index of 37 tomato introgression lines. Table S2. Residual biomass and leaf area of tomato introgression lines. (DOCX 113 kb) [file 12863_2016_362_MOESM1_ESM.docx]

**Supplementary Table S1.** Fruit yield and harvest index of 37 tomato introgression lines.

| Introgression  lines | No. fruits per pt | | Fruit weight per pt (g) | | Fruit mean weight (g) | | Harvest index |  |
| --- | --- | --- | --- | --- | --- | --- | --- | --- |
| IL 1-1 | 11.5 | ip | 434.3 | lo | 38.1 | hn | 46.1 | lp |
| IL 1-3 | 9.8 | mp | 441.7 | lo | 44.7 | cf | 38.4 | pr |
| IL 1-4 | 7.7 | p | 319.2 | no | 40.9 | fl | 32.3 | r |
| IL 2-1 | 15.3 | fl | 450.0 | ln | 32.3 | os | 66.7 | be |
| IL 2-2 | 7.8 | p | 348.9 | mo | 42.8 | dh | 42.7 | nq |
| IL 2-5 | 19.7 | cf | 958.2 | ab | 48.5 | c | 53.3 | fm |
| IL 2-6 | 30.0 | b | 764.4 | cf | 29.9 | sv | 40.8 | or |
| IL 3-1 | 37.0 | a | 840.0 | bd | 22.7 | w | 70.0 | bc |
| IL 3-2 | 10.8 | lp | 316.8 | no | 29.9 | sv | 71.3 | ab |
| IL 3-3 | 10.8 | lp | 518.3 | im | 47.5 | cd | 58.5 | eh |
| IL 3-4 | 14.2 | gn | 435.6 | lo | 30.7 | ru | 55.1 | fl |
| IL 3-5 | 11.8 | ip | 293.9 | no | 26.3 | uw | 44.2 | mp |
| IL 4-1 | 15.7 | ei | 589.2 | gl | 39.0 | hm | 43.8 | mp |
| IL 4-3 | 10.1 | mp | 266.1 | o | 26.6 | tw | 55.7 | fl |
| IL 4-4 | 3.3 | pq | 116.7 | op | 35.3 | mr | 11.2 | s |
| IL 5-1 | 9.6 | np | 391.7 | mo | 39.3 | hm | 54.1 | fl |
| IL 5-2 | 17.1 | dh | 760.0 | cf | 44.2 | cg | 68.6 | bd |
| IL 5-3 | 11.0 | ip | 455.6 | ln | 41.8 | ei | 71.6 | ab |
| IL 6-1 | 20.1 | ce | 748.7 | cg | 37.1 | io | 54.4 | fl |
| IL 6-2 | 2.3 | q | 25.0 | p | 11.1 | *z* | 9.1 | s |
| IL 6-3 | 15.5 | el | 977.8 | ab | 62.7 | a | 51.7 | gn |
| IL 7-1 | 18.0 | dg | 686.7 | dh | 35.8 | lq | 79.9 | ab |
| IL 7-2 | 22.8 | c | 777.8 | cf | 34.0 | ns | 47.6 | ip |
| IL 7-4 | 8.7 | op | 377.0 | mo | 44.2 | cg | 33.9 | qr |
| IL 8-1 | 9.6 | np | 288.3 | no | 30.0 | sv | 56.6 | fi |
| IL 8-2 | 9.9 | mp | 344.5 | mo | 33.9 | ns | 46.0 | lp |
| IL 8-3 | 21.3 | cd | 822.2 | be | 39.0 | hm | 62.7 | bf |
| IL 9-1 | 17.2 | dh | 523.6 | hm | 31.5 | pt | 52.8 | fm |
| IL 9-2 | 32.2 | b | 986.7 | ab | 31.0 | qu | 53.3 | fm |
| IL 9-3 | 17.0 | dh | 465.3 | ln | 29.5 | sv | 43.9 | mp |
| IL 10-1 | 9.1 | op | 360.8 | mo | 36.2 | lp | 50.4 | ho |
| IL 10-2 | 18.7 | cg | 866.7 | bc | 46.1 | ce | 56.6 | fi |
| IL 10-3 | 14.5 | gm | 635.5 | fi | 39.7 | gm | 68.8 | bd |
| IL 11-1 | 15.4 | el | 423.8 | lo | 25.5 | vw | 54.3 | fl |
| IL 11-2 | 18.0 | dg | 507.8 | im | 29.0 | sv | 59.6 | dh |
| IL 11-3 | 11.8 | ip | 429.2 | lo | 35.2 | mr | 62.6 | bf |
| IL 12-4 | 19.5 | cf | 1043.1 | a | 53.8 | b | 57.1 | ei |
| M82 (contr) | 17.5 | dh | 665.0 | ei | 38.0 | hn | 61.5 | cg |

No. (number), pt (plant). d.w. (dry weight), f.w. (fresh weight). Within each column, means followed by different letters are significantly different according to the Duncan test at p≤0.05.

**Supplementary Table S2.** Residual biomass and leaf area of tomato introgression lines.

| Introgression  lines | Biomass per pt  g f.w. pt^-1^ | | Biomass per pt  g d.w. pt^-1^ | | Leaf area  m^2^ pt^-1^ | |
| --- | --- | --- | --- | --- | --- | --- |
| IL 1-1 | 506.1 | fh | 83.3 | fh | 0.47 | r |
| IL 1-3 | 705.6 | ce | 117.0 | ce | 1.85 | ln |
| IL 1-4 | 680.8 | ce | 111.4 | ce | 2.04 | lm |
| IL 2-1 | 222.2 | np | 36.5 | np | 1.86 | ln |
| IL 2-2 | 467.8 | fl | 76.7 | fl | 1.56 | mo |
| IL 2-5 | 841.7 | bc | 139.5 | bc | 3.78 | de |
| IL 2-6 | 1136.1 | a | 186.0 | a | 6.60 | a |
| IL 3-1 | 360.0 | ho | 59.8 | go | 1.04 | pq |
| IL 3-2 | 127.8 | p | 21.1 | p | 1.29 | oq |
| IL 3-3 | 363.3 | ho | 60.1 | go | 4.09 | cd |
| IL 3-4 | 377.8 | go | 62.0 | go | 2.33 | il |
| IL 3-5 | 325.3 | ho | 54.3 | ho | 1.38 | np |
| IL 4-1 | 825.0 | bc | 136.0 | bc | 3.29 | fg |
| IL 4-3 | 210.0 | op | 34.6 | op | 1.00 | pq |
| IL 4-4 | 950.0 | b | 157.7 | b | 2.40 | il |
| IL 5-1 | 296.7 | ip | 48.9 | ip | 1.29 | oq |
| IL 5-2 | 360.0 | ho | 59.9 | go | 2.32 | il |
| IL 5-3 | 202.8 | op | 33.4 | op | 1.55 | mo |
| IL 6-1 | 636.7 | df | 103.8 | df | 3.03 | fh |
| IL 6-2 | 250.0 | mp | 41.3 | mp | 2.79 | gh |
| IL 6-3 | 933.3 | b | 156.6 | b | 1.84 | ln |
| IL 7-1 | 219.4 | np | 36.0 | np | 0.95 | pq |
| IL 7-2 | 835.8 | bc | 138.2 | bc | 2.23 | il |
| IL 7-4 | 760.7 | bd | 124.6 | cd | 3.03 | fh |
| IL 8-1 | 251.3 | mp | 41.6 | mp | 0.82 | qr |
| IL 8-2 | 388.9 | go | 63.9 | go | 2.25 | il |
| IL 8-3 | 480.0 | fi | 79.6 | fi | 3.17 | fg |
| IL 9-1 | 475.8 | fi | 77.7 | fl | 2.64 | hi |
| IL 9-2 | 547.8 | eg | 88.9 | eg | 2.87 | gh |
| IL 9-3 | 695.8 | ce | 115.8 | ce | 4.31 | bc |
| IL 10-1 | 294.2 | ip | 48.7 | ip | 1.66 | mo |
| IL 10-2 | 796.1 | bd | 130.8 | bd | 3.13 | fg |
| IL 10-3 | 286.3 | lp | 47.5 | lp | 1.44 | np |
| IL 11-1 | 315.0 | io | 52.3 | io | 1.18 | oq |
| IL 11-2 | 407.3 | gn | 67.1 | gn | 3.51 | ef |
| IL 11-3 | 292.5 | ip | 48.2 | lp | 1.60 | mo |
| IL 12-4 | 758.1 | bd | 126.6 | cd | 4.55 | b |
| M82 (contr) | 420.0 | gm | 69.8 | gm | 1.62 | mo |

f.w. (fresh weight), d.w. (dry weight), pt (plant). Within each column, means followed by different letters are significantly different according to the Duncan test at p≤0.05.
