# Supplementary figures and images for: Exploring tomato Solanum pennellii introgression lines for residual biomass and enzymatic digestibility traits
Source: BMC Genet. 2016 Apr 5;17:56. doi: 10.1186/s12863-016-0362-9 (PMC4820949; doi:10.1186/s12863-016-0362-9)

Water pretreatment

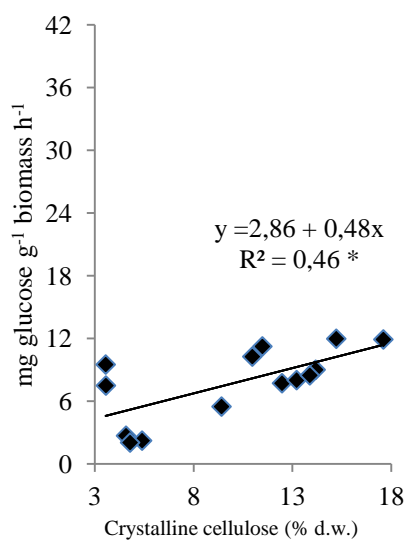

Acid pretreatment

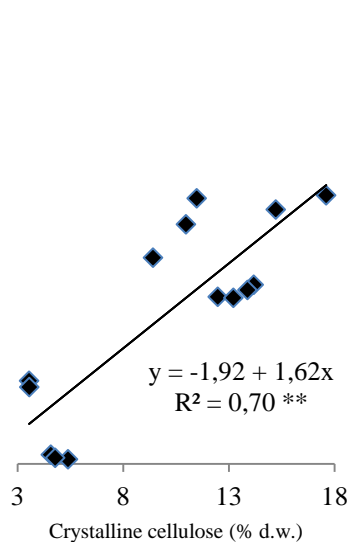

Alkali pretreatment

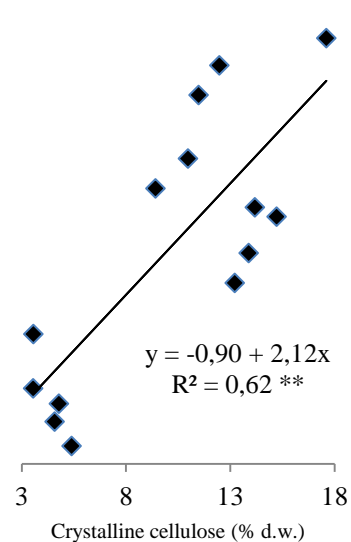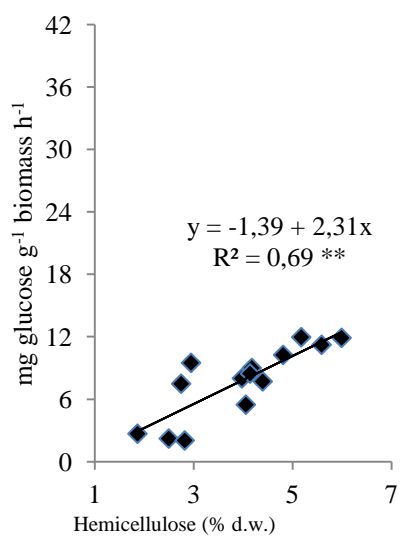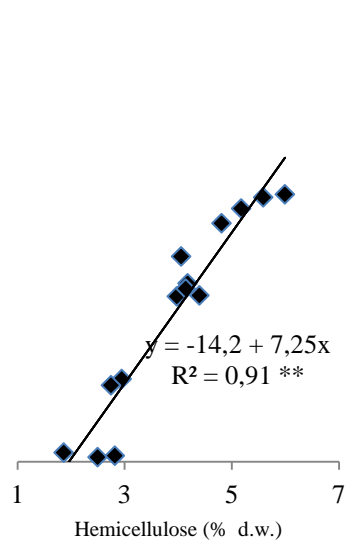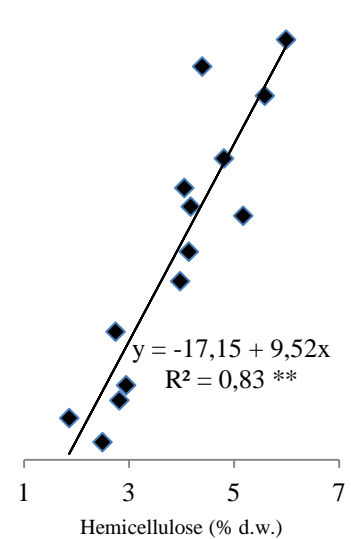

Supplement: Additional file 3: Figure S1. — Correlation between saccharification rate and crystalline cellulose and between saccharification rate and hemicellulose in 13 tomato introgression lines plus M82. (PDF 13 kb) [file 12863_2016_362_MOESM3_ESM.pdf]
